# The Flu Vaccination May Have a Protective Effect on the Course of COVID-19 in the Pediatric Population: When Does Severe Acute Respiratory Syndrome Coronavirus 2 (SARS-CoV-2) Meet Influenza?

**DOI:** 10.7759/cureus.12533

**Published:** 2021-01-06

**Authors:** Anjali Patwardhan, Adrienne Ohler

**Affiliations:** 1 Child Health, University of Missouri, Columbia, USA; 2 Child Health Research Institute, University of Missouri School of Medicine, Columbia, USA

**Keywords:** sars-cov-2, covid-19, viral interference, cross immunoreactivity, influenza vaccine, pneumococcal vaccine, herd immunity

## Abstract

Background

In the midst of the severe acute respiratory syndrome coronavirus 2 (SARS-CoV-2) pandemic, a lot more chaos could be anticipated in the flu season due to the coexistence of SARS-CoV-2 and influenza with almost similar epidemiologic and clinical features. Could this become a "twindemic" or “syndemic” if there is any viral interference occurs? We investigated the effect of influenza and pneumococcal vaccines on the disease course of SARS-CoV-2 in the pediatric population and the possibility of viral interference.

Material and methods

After approval from Institutional Review Board, a retrospective electronic chart review on 20 years and younger SARS-CoV-2 polymerase chain reaction (PCR) positive patients who visited Arkansas Children’s Hospital System between February 1 to August 30, 2020, was performed. The clinical data was collected along with influenza and pneumococcal vaccination status of these patients.

Results

The results showed that viral interference may have played a role in the current flu and coronavirus disease 2019 (COVID-19) twindemic. SARS-CoV-2 and influenza may have significantly affected each other’s epidemiological features.

Conclusion

Understanding the relationship and co-existence of other viruses alongside SARS-CoV-2 and knowing the vaccination status of the host population may help in deploying the right strategies to get the best outcomes.

## Introduction

The flu season is already progressing while the world is still struggling to survive the coronavirus disease 2019 (COVID-19) pandemic. This year, a lot more chaos could be anticipated in the flu season due to the coexistence of severe acute respiratory syndrome coronavirus 2 (SARS-CoV-2) and influenza with almost similar clinical presentations. A more pertinent question is if these two viral infections together can bring a "twindemic" to the planet earth by working synergistically. The flu vaccine has already earned skepticism about the efficacy, misconceptions about the safety, and vaccine-hesitation over the years. The skepticism is reported to be worse in minority populations, so is COVID-19 incidence. The studies suggesting flu vaccination can increase the risk of non-influenza respiratory virus infections further intensify the questions about viral interference [1, 2]. The concerns are that the SARS-CoV-2 virus infection in the individuals vaccinated for influenza can trigger a booster response for flu through memory cells. Simultaneously, the memory cells will inhibit the engagement and activation of naïve B cells by the SARS-CoV-2 virus leading to an inadequate immune response, explaining the increased incidence of COVID-19 after flu vaccination [3]. It has been suggested that due to viral interference in patients with co-infection, the viral shedding of SARS-CoV-2 may get significantly reduced, making detection of the virus in swabs difficult [4].

The second assumption is based on the 'original antigenic sin (OAS) theory.' The OAS suggests that in the human hosts, the first virus antigen exposure (even if it is in the killed vaccine) determines the responses of subsequent viral antigen exposures [5-7]. Some scientists have argued against OAS in influenza infections in animal studies [8, 9]. Another way the two viruses can influence each other is through cross-immunity. We have learned from experience with streptococcal infections that cross-immunity can be detrimental in bacterial infections also. Rheumatic fever, Sydenham chorea, and streptococcal nephritis are glaring examples of diseases due to cross-immune-reactive host [6] and bacterial antigens, and molecular mimicry [10, 11]. 

Research on the pediatric population is critical because though the pediatric population is mostly but not always expected to run a milder disease course, it also makes a larger population group. Therefore, it may play a significant role in influencing viral transmission, the competitive consumers of medical resources, hence an inseparable component of the rampant pandemic. We know that in children, influenza infection itself in the absence of vaccination increases the susceptibility to other serious illnesses (bacterial viral and flare in autoimmune diseases) [12, 13]. So far, no research is available to elaborate on the influence of flu vaccination on pediatric COVID-19. We report from a single center in the USA, our experience in 989 pediatric SARS-CoV-2 positive patients with and without current flu vaccination. We investigated the effect of influenza and pneumococcal vaccines on the disease course of SARS-CoV-2 in the pediatric population. 

## Materials and methods

After approval from Institutional Review Board (IRB #261599), a retrospective electronic chart review was performed on SARS-CoV-2 positive patients to retrieve clinical data and flu vaccination information for the flu season 2019-2020. Since the personal identifying information (PHI) was not included in the analysis and results, a consent waiver was obtained from IRB. All the consecutive patients who tested positive for SARS-CoV-2 were identified by utilizing keywords such as “COVID-19”, “SARS-CoV-2 PCR”,” SARS-CoV-2 RT-PCR”. The patients included all the children aged 20 years or below who visited the University of Arkansas Medical Sciences, Arkansas Children's Hospital Systems (USA) between February 1 to August 30, 2020, for their healthcare needs or were tested due to exposure to the COVID-19 patients at disaster testing mobile sites. The nasal and nasopharyngeal swabs were collected for virus testing. As per the Centers for Disease Control and Prevention (CDC) recommendations, the diagnostic tests used were reverse-transcription polymerase chain reaction (RT-PCR) and polymerase chain reaction (PCR). The patients aged 20 years and below were defined as the pediatric population.

The subjects vaccinated for killed influenza vaccine during the current flu season (September 2019-April 2020) only were considered 'flu-vaccinated for the purpose of this study.' The previous influenza vaccinations were not taken into consideration. The patient was deemed to be vaccinated for the pneumococcal vaccine if he/she had received a full age-appropriate course of pneumococcal vaccine. The vaccination records of 73 patients were not accessible out of 989 patients. An additional 11 subjects had missing records for age, allergies, comorbidities, or sex, so those patients were not included in statistical analysis.

For the analysis, there are two variables: a) symptomatic and b) severity. The severity variable for the logistic analysis was grouped into two categories: 1) asymptomatic and 2) mild, moderate, severe, and critical cases. These two variables (symptomatic and severity) are created somewhat differently (i.e., not feeling well vs. objective symptoms). Thus, that is how someone can be symptomatic but NOT be a mild, moderate, severe, or critical case. The reference case for the logistic regression for both a) and b) definitions are asymptomatic cases.

The symptomatic was defined as anything from patients not feeling well or not feeling him/herself to objective multisystem symptoms and fever. Simultaneously, the severity was defined when the patient had either no symptoms or objective symptoms (mild, moderate, severe, and critical). Therefore, a small group of patients may fall into the symptomatic category without qualifying into any severity categories because they did not have specific signs/symptoms but just did not feel right.

Only the individuals who objectively tested positive for SARS-CoV-2 by RT-PCR and PCR, as listed above, were included in the study. The suspected cases (exposed or symptomatic but not positive) were not included in the study. The severity of infection was categorized as asymptomatic (incidentally detected, no symptoms), mild (mild symptoms, managed at home), moderate (symptomatic, needing less than 24 hours hospital admission or emergency room observation, IV fluids), severe (symptomatic needing hospitalization >24 hours/pediatric intensive care unit [PICU], nasal oxygen, IV fluids), critical (symptomatic, needing artificial ventilation for respiratory failure /acute respiratory distress syndrome [ARDS], had a shock, encephalopathy, heart failure, renal failure, coagulation dysfunction, a multisystem inflammatory syndrome in children [MIS-C], coagulopathy, death).

The final cohort of 905 patients was identified to have complete records for analysis. Reminding the patient classification from analysis purpose, the symptomatic was defined as anything from patients not feeling well or not feeling him/herself to objective multisystem symptoms and fever. The severity of illness was defined in five degrees, such as asymptomatic (patient had no symptoms) or objective symptoms; therefore, will fall into mild, or moderate, or severe, or critical severity category. 

Outcomes variables include three metrics: a) whether the patient was symptomatic (binary), b) whether the patient exhibited respiratory symptoms (binary), and c) the level of severity (binary). Due to low rates of moderate, severe, and critical cases, the severity level is recoded as binary: asymptomatic vs. mild to critical. Pearson's Chi-squared test (categorical variables) or Wilcoxon rank-sum test (quantitative variables) were performed to examine differences between those with and without immunization. In the study cohort, 51.49% had been immunized for the flu. Several significant differences exist between those with and without influenza immunization, including comorbidities (p=0.013), race (p=0.013), whether the child has allergies/asthma (p=0.009), and whether the child had a pneumonia vaccination (p<0.0001), highlighting the importance of adjusting odds ratio (OR) estimates by these covariates. 

To analyze symptoms and severity, logistic regressions were estimated adjusting for confounding bias through the set of control variables: race, sex at birth, age, the month of diagnosis, whether the patient has allergies or asthma if the patient has comorbidities if the child is obese, and whether the patient is regularly exposed to smoke, with robust standard errors. All tests were two-sided, and a p-value of < 0.05 was regarded as statistically significant. All statistical analyses were conducted using STATA software version 16 (STATA Corp., Texas, USA).

## Results

Results in our cohort of the 905 SARS-CoV-2 positive pediatric patients suggest that 62.51% of patients had asymptomatic diseases. Of the patients who had symptomatic infections, 33.20% qualified for mild disease, 3.37% for moderate diseases, 0.61% had a rate of severe illnesses and only 0.31% of critical illnesses as per the criteria adopted from published literature and defined in the methods section. The SARS-CoV-2 positive patients who were vaccinated for influenza in the current flu season were compared with those not vaccinated (Table 1). The previous influenza vaccinations were not taken into account. The results illustrated in Table 2 show that the patients who were vaccinated for influenza had lower odds of having symptomatic diseases than those not vaccinated (p=0.028, adj. OR=0.714, 95% CI [0.529, 0.964]). The SARS-CoV-2 positive patients who were ever fully immunized for the pneumococcal vaccine, and those who were never vaccinated were also compared. The results showed that the patients who are vaccinated for the pneumococcal vaccine had lower odds of having symptomatic disease than those unvaccinated (p=0.010, adj. OR=0.482, 95% CI [0.277, 0.837]) in our cohort (Table 2). 

**Table 1:** Patient characteristics, immunization status and comorbidities

Status/characteristics	Flu immunized (No)	Flu immunized (Yes)	χ^2^ p-value
N (number)	466 (51.49) %	439 (48.51%)	
Symptomatic			
Yes	170 (36.48%)	129 (29.38%)	0.023
No	296 (63.52%)	310 (70.62%)	
Respiratory symptoms			
Yes	134 (28.82%)	97 (22.10%)	0.021
No	331 (71.18%)	342 (77.90%)	
Severity			
Asymptomatic	283 (60.73%)	307 (69.93%)	0.004
Symptomatic (mild-critical)	183 (39.27%)	132 (30.07%)	
Covariates			
Sex			
Male	222 (47.64%)	222 (50.17%)	0.378
Female	244 (52.36%)	217 (49.43%)	
Race			
White/Asian/other	128 (27.47%)	157 (35.76%)	0.013
Black	175 (37.55%)	132 (30.07%)	
Hispanic	163 (34.98%)	150 (34.17%)	
Pneumonia immunized			
Yes	411 (88.20%)	432 (98.41%)	0.000
No	55 (11.80%)	7 (1.59%)	
Allergy/asthma			
Yes	99 (21.24%)	126 (28.70%)	0.009
No	367 (78.76%)	313 (71.30%)	
Comorbidities			
Yes	128 (27.47%)	154 (35.08%)	0.013
No	338 (72.53%)	285 (64.92%)	
Obese			
Yes	118 (25.32%)	124 (28.25%)	0.321
No	348 (74.68%)	315 (71.75%)	
Smoke exposed			
Yes	43 (9.23%)	42 (9.57%)	0.861
No	423 (90.77%)	397 (90.43%)	
Wilcoxon p-value			
Age (years) mean (SD)	8.69 (6.05)	8.14 (5.38)	0.399
Month of diagnosis mean (SD)	7.09 (0.80)	7.12 (0.78)	0.613

**Table 2:** Adjusted odds ratio for immunized vs. unimmunized patients Covariate adjustments include race, sex at birth, age, the month of diagnosis, whether the patient has allergies or asthma, if the patient has comorbidities, if the child is obese, and whether the patient is regularly exposed to smoke, with robust standard errors. OR - odds ratio

Vaccination type	OR	p-value	95% CI
Symptomatic			
Influenza	0.714	0.028	(0.529, 0.964)
Pneumonia	0.482	0.01	(0.277, 0.837)
Respiratory symptoms			
Influenza	0.678	0.018	(0.492, 0.934)
Pneumonia	0.765	0.367	(0.428, 1.368)
Severity (mild to critical case)			
Influenza	0.672	0.008	(0.500, 0.903)
Pneumonia	0.412	0.002	(0.234, 0.725)

The patients who were vaccinated for influenza had lower odds to have respiratory symptoms than those not vaccinated (p=0.018, adj. OR=0.678, 95% CI [0.492, 0.934]). However, the results fail to show a difference between the vaccinated and unvaccinated patients for the pneumococcal vaccine in terms of respiratory symptoms.

Finally, the patients who were vaccinated for influenza had lower odds of having severe diseases (mild to critical) than those not vaccinated (p=0.008, adj. OR=0.672, 95% CI [0.500, 0.903]), and those vaccinated for pneumonia also had lower odds of severe diseases (mild - critical) than those not vaccinated (p=0.002, adj. OR=0.412, 95% CI [0.234, 0.725]).

Additionally, we report that as time went on and the pandemic evolved, patients were more likely to be asymptomatic (p=0.006), less likely to have respiratory symptoms (p=0.037), less likely to fall into symptomatic categories of patients (ranging from mild- critical) (p=0.004). This result likely reflects the changes in testing requirements and the tested population from June to August, as testing capacity increased. The effect of growing herd immunity or possible viral mutation can only be an assumption without data evidence at this point.

Table 2 shows that those with comorbidities were more likely to exhibit symptoms (p=0.000, adj. OR=1.13, 95% CI [1.32-2.47]), have respiratory symptoms (p=0.001, adj. OR=1.74, 95% CI [1.25, 2.42]), and have greater odds of severe disease course (p=0.001, adj. OR=1.71, 95% CI 1.25, 2.34]).

Black children had smaller odds than White/Asian/Other children to exhibit symptoms (p=0.037, adj. OR=0.67, 95% CI [0.463, 0.977]), and were less likely to be symptomatic (mild-critical) case (p=0.029, adj OR=0.66, 95% CI [0.458, 0.959]), despite of comparatively higher positivity rate.

## Discussion

It is known that the growth of one virus can be inhibited by the previous infection with another (related or unrelated) virus in the same host through mediators like interferons and others. The phenomenon is called virus interference. The virus interference can occur even when the first virus invader is an inactivated virus, such as in the vaccines. The human hosts generate interferons after the encounter with their first viral antigen. The interferons then attach to the other cell receptors and modulate cellular structure and function to protect from further infections. The first virus interferes with the invasion from the second virus in complex ways. The first virus may achieve virus interference by inducing protective mechanisms in the host immune system or inhibiting replication through competitive survival (auto interference), competitive binding to B-cells, or inhibit activation of naïve B cells by the second viral infection. The interference may occur by damaging/modifying the host's cellular receptors or enzymes required for the attachment, multiplication, replication, and survival of the second virus in the host cells. There may occur a transient (1-2 weeks) immunity for a second virus infection after the first viral encounter. The memory cells may respond to the second virus by increased antibodies against the first viral antigen coupled with decreased antibody production against second viral invaders, increasing the risk for severe disease by the second virus. Different viruses show different virus interference levels, e.g., the influenza A(H1N1) pdm09 virus was reported to be the most potent inhibitor of subsequent viral infections [14]. Conversely, it was reported in the elderly population during the 2009 H1N1 pandemic that pre-infection seasonal H1N1 virus antibodies cross-reacted and neutralized pandemic influenza A(H1N1) pdm09 virus [15]. In the past, a transient (1-2 weeks) viral interference was reported in children after flu and polio vaccination, followed by an increase in non-flu respiratory and gastrointestinal infections [1, 16].

It will be interesting to see if changes in the flu infection rate occur in the current flu season due to SARS-coV-2 induced viral interference. The CDC influenza surveillance system is already reporting a lower influenza infection incidence this year [17]. In week 44/52 (ending October 31, 2020) this year, as per the Influenza-like Illness Surveillance Network (ILINet), only 1.3% of patient outpatient visits were reported than the national baseline of 2.6%. Although, the National Center for Health Statistics (NCHS) Mortality Surveillance has reported an increase in the cumulative deaths for pneumonia, COVID-19, and influenza for the 44/52 week to 8.1%, which is significantly above the previous epidemic thresholds (6.0%) for the same time of the year [17]. The lower incidence of influenza may well be explained by social distancing, lockouts, and mask-wearing. We do not have enough information to comment on viral interference causing a decrease in influenza incidence yet.

Our results confirm the finding from several previous studies in the pediatric population that patients with comorbidities and obesity run a higher risk for suffering from symptomatic and severe diseases [18, 19].

Interestingly, African American patients represented 37.5% of all influenza vaccinated, and 30% of all pneumococcal vaccinated SARS-CoV-2 positive patients. Despite the comparatively higher positivity rate, the relative incidence (while vs. Hispanic and Asian) of symptomatic and severe diseases was lower in African American patients. This finding is different from some other published reports on the pediatric population [20]. This finding is the first time reported in the pediatric population.

The recent comments published in Lancet also agree that COVID-19 is not a pandemic but a 'syndemic' because several dimensions in the environment, virus, and the host will influence the disease outcome and spread [21].

Several researchers have reported that on exposure to the second (related or unrelated) virus, the antibody response to the new virus was better in the naïve individuals than those who had repeated flu vaccinations or exposure to the flu virus in previous years [22].

It is too early to say if our results can be explained by viral interference alone or have more than a few explanations for the protective value of flu vaccination against SARS-CoV-2 infection. The flu vaccine prevents the flu to a reasonable extent, and the unvaccinated population is more likely to get active influenza diseases. If falling sick with influenza makes patients more susceptible to SARS-coV-2 infection is yet to be seen. The extent of social distancing (at least less socializing), lockouts, and restrictive lifestyle during this flu season will undoubtedly influence influenza infection rates producing bias and confounding factors.

A group of researchers has reported the beneficial effect of flu vaccination on SARS-CoV-2 related mortality in the elderly (65 years and above) population [23]. But no such study in the pediatric population is available to our knowledge.

As per, U.S. Department of Health and Human Services (HHS), Office of Minority Health (OMH), the minority population is less likely to be vaccinated for flu [24].

## Conclusions

Seasonal influenza and pneumococcal vaccination may have protective value in symptomatic COVID-19 diseases. In our mixed population cohort, the patients were less likely to develop symptomatic and severe infections if they had received seasonal influenza vaccination in the current flu season and were vaccinated for pneumococcal vaccines. The serial and past influenza vaccination status was not included in the analysis. Our study results point to the possibility of viral interference and stress its significant public health implications in the current situation. The "twindemic" of flu and SARS-CoV-2 may become a public health nightmare because of the same seasonal occurrence and similarity in symptoms apart from interactions between the viruses and their host responses. There is an urgent need to explore how two viruses' coexistence affects the individual virus's epidemiology and host responses in a larger geographical-multiracial and prospective cohort. The fall time marked the influenza-SARS-CoV-2 season while the schools open, making the pediatric population a top priority for the researchers. Based on our observations, we hypothesize that the higher incidence of COVID-19 in minority populations may also reflect their low influenza-pneumococcal vaccination rate apart from health inequalities. Understanding the relationship and co-existence of other viruses alongside SARS-CoV-2 and knowing the vaccination status of the host population may help in deploying the right strategies to get the best outcomes.

Our study also confirms the findings of previous reports informing the patients with comorbidities are more likely to be symptomatic, have respiratory symptoms, and have greater odds of severe disease course. In our cohort, the African American children had smaller odds than White/Asian/other children to exhibit symptoms or have severe diseases. They were less likely to have severe diseases course despite a comparative higher positivity rate than Whites, Hispanics, and Asians. This finding is the first time reported in pediatric populations. 
